# Surface Roughness Detection of Arteries via Texture Analysis of Ultrasound Images for Early Diagnosis of Atherosclerosis

**DOI:** 10.1371/journal.pone.0076880

**Published:** 2013-10-17

**Authors:** Lili Niu, Ming Qian, Wei Yang, Long Meng, Yang Xiao, Kelvin K. L. Wong, Derek Abbott, Xin Liu, Hairong Zheng

**Affiliations:** 1 Paul C. Lauterbur Research Center for Biomedical Imaging, Institute of Biomedical and Health Engineering, Shenzhen Institutes of Advanced Technology, Chinese Academy of Sciences, Shenzhen, China; 2 Centre for Biomedical Engineering, and School of Electrical and Electronic Engineering, University of Adelaide, Adelaide, Australia; University of Washington School of Medicine, United States of America

## Abstract

There is a strong research interest in identifying the surface roughness of the carotid arterial inner wall via texture analysis for early diagnosis of atherosclerosis. The purpose of this study is to assess the efficacy of texture analysis methods for identifying arterial roughness in the early stage of atherosclerosis. Ultrasound images of common carotid arteries of 15 normal mice fed a normal diet and 28 apoE^−/−^ mice fed a high-fat diet were recorded by a high-frequency ultrasound system (Vevo 2100, frequency: 40 MHz). Six different texture feature sets were extracted based on the following methods: first-order statistics, fractal dimension texture analysis, spatial gray level dependence matrix, gray level difference statistics, the neighborhood gray tone difference matrix, and the statistical feature matrix. Statistical analysis indicates that 11 of 19 texture features can be used to distinguish between normal and abnormal groups (p<0.05). When the 11 optimal features were used as inputs to a support vector machine classifier, we achieved over 89% accuracy, 87% sensitivity and 93% specificity. The accuracy, sensitivity and specificity for the k-nearest neighbor classifier were 73%, 75% and 70%, respectively. The results show that it is feasible to identify arterial surface roughness based on texture features extracted from ultrasound images of the carotid arterial wall. This method is shown to be useful for early detection and diagnosis of atherosclerosis.

## Introduction

Diagnosis of atherosclerosis in its early stage is important for predicting the occurrence of strokes and heart attacks, and provides opportunities for their prevention. The earliest pathological abnormality in atherosclerosis is the fatty streak, which is an accumulation of lipid-filled macrophages within the intima of the artery [1]. To predict the silent progression of atherosclerosis more effectively, various studies based on blood fluid and cardiovascular wall mechanics have been undertaken to identify vessel abnormalities prior to end organ damage [2], [3]. The examination of the carotid arteries is of high clinical interest since the carotid and cerebral vessels have significant implications for stroke. The measurement of carotid intima-media thickness (IMT) has become an accepted and reliable surrogate marker for identification of atherosclerosis [4], [5]. However, recent studies have shown that it is highly likely that the inner surface of the arterial wall becomes rough before becoming thicker [6], [7]. Thus, it is potentially useful to evaluate roughness of the inner structures of the arterial wall for early diagnosis of atherosclerosis.

Ultrasound is one of the most widely used techniques for non-destructive testing of roughness. Wilhjelm et al. developed an angular spectrum-based formulation method to study planar rough phantoms [8]. As the surface roughness varies significantly, the relative proportions of specular reflection and scattering vary correspondingly. The higher the surface roughness is, the higher the proportion of scattered ultrasound becomes. As such, Gunarathne et al. developed an ultrasonic spectroscopic technique to measure the gross surface texture of non-medical materials. They introduced a roughness coefficient to specify the texture on a quantitative basis [9]. Then, Schmidt-Trucksäss et al. proposed the arithmetic mean of the IMT profile deviations to characterize the roughness of common carotid arteries [6]. Later on, Zhang et al. developed a 3D ultrasound system to extract surfaces using radial basis functions. This method produced realistic surfaces with a high level of detail [10]. But the measurement error lies in the millimeter range. To detect minute roughness, Cinthio et al. utilized the phase change that occurs in a radio frequency (RF) echo from the rough surface of an object during its lateral motion [7]. However, their method requires the acquisition of high frame rate RF data.

Image processing methods have potential in providing the objective and quantitative evaluation of arterial roughness. Our previous studies and the other existing works have identified texture analysis as being useful in the analysis of ultrasound images [11]–[13]. To date, plaque texture analysis has been applied to conventional 2D brightness mode (B-mode) ultrasound images and has been shown to have considerable success in identifying symptomatic carotid plaques including risk stratification and predicting stroke plaques. First-order statistics, especially the gray scale median (GSM), were widely used in the study of plaque echogenicity [14]. Low GSM values were associated with symptomatic plaques, high degrees of stenosis and a high incidence of ipsilateral brain infarction. Second-order statistics were estimated from B-mode ultrasound images of the carotid artery and correlated with plaque histology [15]. The fundamental laws of texture energy were used to automatically classify plaques into symptomatic or asymptomatic classes [16]. The fractal dimension was found to be able to differentiate between symptomatic and asymptomatic plaques [17]. In addition, Christodoulou et al. showed that texture features, shape parameters, and morphological features from carotid plaques could be applied to discriminate subjects with symptomatic and asymptomatic stenosis [18]. Loizou et al. utilized texture analysis to evaluate ten despeckle filters that are used to improve the class separation between symptomatic and asymptomatic plaques [19]. In addition, Tsiaparas et al. proposed a multiresolution approach to discriminate between symptomatic and asymptomatic cases [20]. Recently, 3D carotid imaging with plaque texture feature analysis was adopted for the study of asymptomatic carotid stenosis and a risk of stroke study [21].

All of these previous works underscored the potential of using texture features to evaluate carotid atherosclerotic plaque. To the best of our knowledge, no previous work has used texture analysis to evaluate arterial roughness in the early stage of atherosclerosis. Therefore, the aims of this study are: (1) to assess the efficacy of texture analysis methods in identifying arterial roughness; and (2) to determine which texture features provide a more accurate evaluation.

## Methods

### A. Data Acquisition

A group of 40 apoE^−/−^ C57BL/6 mice (8 weeks of age; Peking University, Beijing, China) were fed with a high-fat diet for 16 weeks. Twenty 8-week-old normal C57BL/6 mice were fed a normal chow diet as control group. In order to standardize the ultrasound scanning protocol, only 28 apoE^−/−^ mice and 15 normal mice with heart rate within the range of 300–500 bpm during anesthesia were included for texture analysis. All animal experiments were conducted in accordance with the protocol approved by the Institutional Animal Care and Use Committee of Shenzhen Institutes of Advanced Technology, Chinese Academy of Sciences.

Ultrasound data were acquired using a high-frequency ultrasound system (Vevo 2100, Visualsonics, Toronto, Canada) equipped with a linear array transducer (MS 550D, frequency 22–55 MHz). The mouse was first anesthetized with an intraperitoneal injection of 50 mg/kg pentobarbital sodium (1% in normal saline) and anesthesia was sustained with 1.5% isoflurane (delivered in 100% O_2_) supplied through a nose cone. The mouse was then placed on a heated procedure board and the limbs were gently taped to ECG electrodes coated with electrode cream and a rectal thermometer was inserted for maintenance of normothermia (37°C internal temperature). The imaging location, on each mouse, was carefully shaved of any fur, and warmed ultrasound gel was liberally applied to ensure optimal image quality. The left common carotid artery (CCA) was imaged and visualized in a long-axis view and a CINE loop of 100 frames was stored for later off-line analysis. The time gain compensation curve was adjusted (gently sloping) to produce uniform intensity of echoes on the screen. The gain was set to 30 dB and the dynamic range to 65 dB. To reduce variability, image parameters remained constant throughout the experiment (i.e., focus and depth optimized for each animal at the beginning of the experiment and the point of monitoring was fixed through the entire experiment). All examinations were performed by one experienced operator and all the measurements were repeated three times at the same site.

Because B-mode ultrasound images of the carotid artery are characterized mainly by two brightness levels, a darker one corresponding to blood and a brighter one corresponding to surrounding tissue, a region of interest (ROI) proximal to the carotid bulb can be manually selected not to include accidently some surrounding tissue by the operator for image post-processing. A total of 133 CCA-ROIs (45 normal and 88 abnormal) obtained from 15 normal and 28 abnormal mice, were analyzed. Fifteen normal and twenty-eight abnormal CCA-ROIs were used to train the classifier, and the remaining 30 normal and 60 abnormal CCA-ROIs were applied to evaluate the classifier.

### B. Histology

Immediately after all the imaging procedure has been completed, the mice were euthanatized. Then, the CCA samples were dissected for histologic phenotyping, covered with Tissue-Tek (Sakura, Torrance, CA, USA), and frozen in liquid nitrogen vapor. The CCA sections (5 µm thick) were cut with a cryostat microtome (CM1950, Leica, Heidelberg, Germany) and were routinely stained with hematoxylin and eosin (H&E). Tissue sections were viewed under a confocal microscope (FV1000, Olympus, Tokyo, Japan).

### C. Feature Extraction

Texture features were extracted from the ROI for identifying arterial roughness. Textures are characteristic intensity variations that typically originate from roughness of object surfaces [22], and contain important information about the structural arrangement of image content and surfaces. In this paper, a total number of 19 texture features were extracted from the ROIs. The tested feature sets have also been successfully used in previous work in texture analysis [18], [23]. Some of the used features (energy and entropy; coarseness and busyness) capture complementary textural properties. Features that did not reach statistical significance were eliminated. Six different texture feature sets (a total of 19 features) are described in detail as follows:

#### 1) First-Order Statistics (FOS)

The FOS describes the gray level histogram of an image (or of some local ROI of an image). Statistical properties of the gray level histogram are frequently used for texture description. In this study, two texture features, namely, average gray level (AGL) and standard deviation (SD) were computed from 43 CCAs (15 normal CCAs and 28 abnormal CCAs). Here, the AGL gives the measure of average gray level; SD measures how spread out the gray levels are around the AGL,
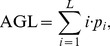
(1)

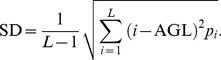
(2)


Here, *p_i_* is the probability of occurrence of gray level value *i*, and *L* is the number of possible gray levels.

#### 2) Fractal Dimension Texture Analysis (FDTA)

Mandelbrot developed the fractional Brownian motion model to describe the roughness of natural surfaces [24]. The Hurst coefficient *H* ^(*w*)^ was computed [25] for image resolutions *w = *1, 2, 3. Fractal dimension D_f_ can be computed from the relationship
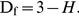
(3)


A small value of D_f_ (large value of parameter *H*) means a smooth surface, and large D_f_ (small *H*), a rough surface.

#### 3) Spatial Gray-Level Dependence Matrix (SGLDM)

The SGLDM is a statistical method which constructs co-occurrence matrix to reflect the spatial distribution of gray levels in the ROI [26]. It is based on the estimation of the second-order joint conditional probability density functions, *f*(*i*, *j*; *d*, θ). Each *f*(*i*, *j*; *d*, θ) is the probability of going from gray-level *i* to gray-level *j*, given that the intersample spacing is *d* and the direction is specified by the angle *θ*. The estimated values for these probability density functions will be denoted by *P*(*i*, *j*; *d*, *θ*).

Haralick et al. proposed 14 measures that can be employed to extract useful texture information from *P*(*i*, *j*; *d*, *θ*) [26]. In this study, we used five measures as follows:

- Contrast: It is a measure of the local variations of gray levels present in an image. Images with large neighboring gray level differences are associated with high contrast. This parameter can also characterize the dispersion of the matrix values from its main diagonal. Note that the contrast is defined as follows:
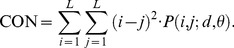
(4)
- Correlation: It is a measure of gray level linear-dependencies in an image. High correlation values (close to 1) imply a linear relationship between the gray levels of pixel pairs. This correlation is defined as follows:
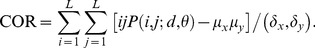
(5)where *µ_x_* and *σ_x_* are the mean and standard deviation of the row sums of the matrix *P*(*i*, *j*; *d*, *θ*), and *µ_y_* and *σ_y_* are the corresponding statistics of the column sums.
- Energy: It is a measure of image homogeneity and reflects pixel-pair repetitions. Homogeneous images have few dominant gray tone transitions, which result in higher energy. The energy is defined as follows:
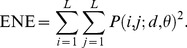
(6)
- Homogeneity: It is a measure of local homogeneity in an image. It assigns larger values to smaller gray level differences within pixel pairs. This parameter gets higher when the texture includes more homogeneous regions. The homogeneity is written as:
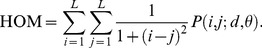
(7)
- Entropy: It is a measure of non-uniformity in an image. If the image is heterogeneous, many elements on *P*(*i*, *j*; *d*, *θ*) have small values, which imply that entropy is very large. The entropy is inversely correlated to energy. It is given by the following equation:
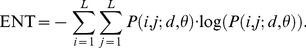
(8)For a chosen distance *d* (in this paper *d* = 1) and for angles 0°, 45°, 90° and 135° we computed four values for each of the above 5 texture measures.

#### 4) Gray Level Difference Statistics (GLDS)

The GLDS algorithm uses FOS of local property values based on absolute differences between pairs of gray levels or of average gray levels [27]. For any given displacement γ = (Δx, Δy), let I_γ_(x, y) = |I(x, y)−I(x+Δx, y+Δy)|. Let f_γ_ be the probability density of I_γ_(x, y). If there are L gray levels, this has the form of an L-dimensional vector whose ith component is the probability that I_γ_(x, y) will have value i. If the picture I is discrete, it is easy to compute f_γ_ by counting the number of times each value of I_γ_(x, y) occurs, where Δx and Δy are integers. Four measures are listed as follows:

- Contrast: It is the second moment of *f*_γ_, whereby it is denoted by:
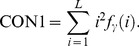
(9)
- Angular Second Moment: It is small when the *f*_γ_ (*i*) are very similar and large when some values are high and others low, e.g., when the values are concentrated near the origin. This is denoted by:
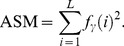
(10)
- Entropy: It is large for equal *f*_γ_ (*i*) and small when they are very unequal. It expressed as:
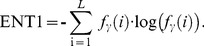
(11)
- Mean: It is small when the *f*_γ_ (*i*) are concentrated near the origin and large when they are far from the origin and is shown by:
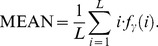
(12)The above features were calculated for displacements γ = (0, 3), (3, 3), (3, 0), (3, −3), where γ = (Δ*x*, Δ*y*), and their mean values were taken.

#### 5) Neighborhood Gray Tone Difference Matrix (NGTDM)

Here, the NGTDM defines features related to human perception of a texture [28]. A NGTDM, *s*(*i*), is a column matrix formed by summing the absolute value of the pixel being observed minus the average of the pixels in its neighborhood. The neighborhood was predefined as a distance of *d = *2 pixels. Once the NGTDM was formed, five texture features were calculated, namely, the coarseness, contrast, busyness, complexity, and texture strength, as follows:

- Coarseness: It is the reciprocal of normalized sum of the deviations of pixel intensities from their neighborhood average intensities. Large values represent areas where gray-level differences are small, i.e., coarse texture. This parameter is expressed as:
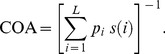
(13)
- Contrast: Perceptually, an image is said to have a high level of contrast if areas of different gray levels are clearly visible. Thus high contrast means that the intensity difference between neighboring regions is large. Also the spatial frequency of the changes in intensity will affect the contrast of an image. The contrast is denoted by:
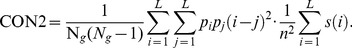
(14)where *N_g_* is the total number of different gray levels in the image. For an *N*×*N* image, *n* = *N*-2*d*.
- Busyness: A busy texture is one in which there are rapid changes of intensity from one pixel to its neighbor; that is the spatial frequency of intensity changes is very high. If these changes are very small in magnitude, they may not be visually noticeable and a high level of local uniformity in intensity may be perceived. Busyness is expressed as:
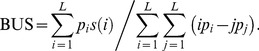
(15)
- Complexity: It refers to the visual information content of a texture. A texture is considered complex if the information content is high. The complexity is given by:
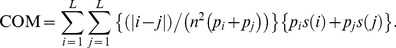
(16)
- Texture Strength: It is a difficult concept to define concisely. However a texture is generally referred to as strong when the primitives that comprise it are easily definable and clearly visible. This parameter is written as:
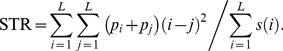
(17)

The SFM is a method that directly evaluates the statistical features for several intersample spacing distances from the image. The following two features, contrast and covariance, are used in this paper are defined as:
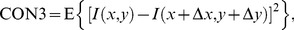
(18)

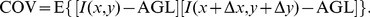
(19)where E(⋅) denotes the expectation operation.

### D. Classifier Design

#### 1) K-Nearest-Neighbor (KNN) classifier

The KNN classifier is used to classify whether a CCA is normal or abnormal. In the KNN algorithm, in order to classify a new input pattern, its *k* nearest neighbors from the training set are identified [29]. A new pattern is classified to the most frequent class among its neighbors based on a similarity measure that is usually the Euclidean distance. The shorter the inter-distance, the greater the similarity. In this paper, the KNN classifier was implemented for *k* = 5.

#### 2) Support Vector Machine (SVM) classifier

The SVM is a relatively new type of learning algorithm, originally introduced by Vapnik and successively extended by a number of other researchers [30]–[32]. The goal of the SVM is to produce a model (based on the training data) that predicts the target values of the test data given only the test data attributes.

Given a training set of instance-label pairs (X*_i_*, C*_i_*), *i* = 1, 2,…, *l* where *l* is the dimension of training set and C∈{−1,1}. The SVM requires the solution of the following optimization problem:
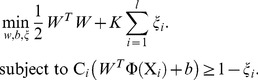
(20)


Here training vectors X*_i_* are mapped into a higher (maybe infinite) dimensional space by the function 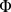. *ξ_i_* ≥0 is the so-called slack variables that allow for misclassification of noisy and difficult data points. The SVM finds a linear separating hyperplane with the maximal margin in this higher dimensional space. Here, *K* >0 controls the tradeoff between the slack variable penalty and the margin. The function 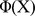 maps the data to a higher dimensional space. This new space is defined by its kernel function that is expressed as 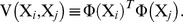


The important advantage of a SVM classifier is the transformation of the learning task to the sequential minimal optimization with linear constraints. For this type of optimization there exist many highly effective learning algorithms [33], [34], leading in almost all cases to the global minimum of the cost function.

### E. Statistical Analyses

Data are expressed as the mean value ± standard deviation. Comparison of continuous variables was performed using Student’s t-test. Significance corrections for multiple comparisons over all features were done using false discovery rate (FDR) correction (p<0.05) [35]. Accuracy, sensitivity and specificity were calculated for each classifier. The accuracy is defined as the percentage of the correctly classified CCAs to the total number of the tested CCAs. Sensitivity (true-positive rate) is the percentage of abnormal mice based classification approach; specificity (true-negative rate) is the proportion of the normal mice based classification approach. One minus specificity is the false-positive rate (1-specificity). Receiver operating characteristic (ROC) curves plot sensitivity VS (1-specificity). Curves toward the upper left-hand corner of a receiver operating characteristic graph represent stronger screening tests. The areas under receiver operating characteristic curves (AUROCs) are used to determine the ability of the KNN and SVM classifiers to distinguish between normal and abnormal CCAs. Higher AUROC corresponds to stronger performance of screening tests. Statistical analyses were performed using SPSS for Windows 17.0 (SPSS Inc).

## Results

**Figure 1:**
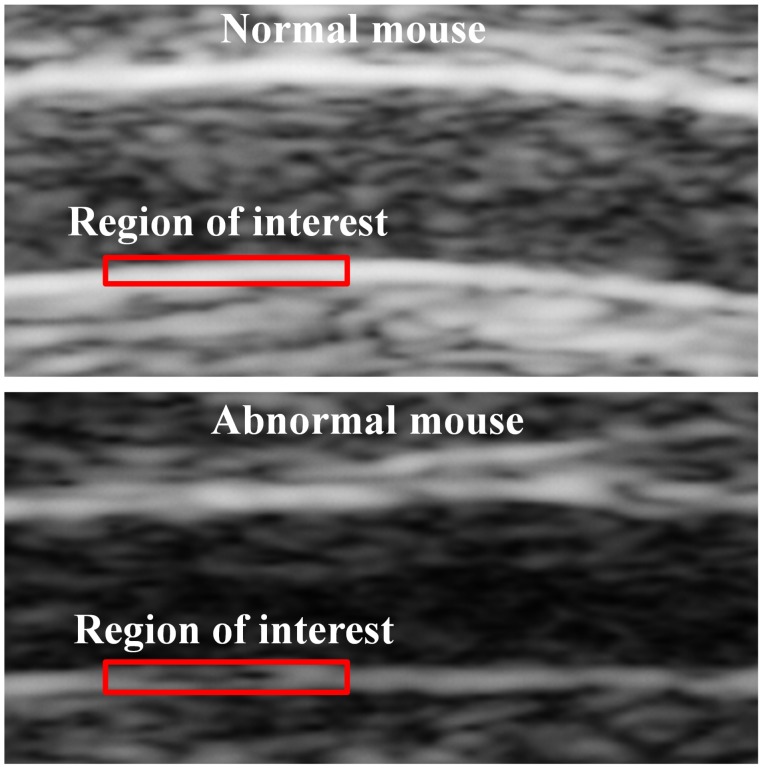
The ultrasound images of the common carotid artery of normal mice fed a normal diet and abnormal mice fed a high-fat diet for 16 weeks, and the regions of interest (ROIs) were outlined in red.

A total of 133 CCA-ROIs were used in this study. One hundred ultrasound images were analyzed for each CCA-ROI. Six different texture feature sets (a total of 19 features) were extracted from the manually segmented CCA images as described in Section II. For each feature set, the mean and standard deviation for the normal and abnormal classes were computed, as well as the p values were obtained using the t-test and were corrected for multiple comparisons by the method of FDR. (Table 1). The 11 texture features were considered to be statistically significant. They are: the average gray level and standard deviation of FOS, the contrast and energy of SGLDM, the contrast and angular second moment of GLDS, the coarseness, busyness, complexity, and texture strength of NGTDM, and the covariance of SFM. Texture in abnormal CCA tends to be more hypoechoic, with higher contrast, more heterogeneous, rougher, coarser and more distinctive, whereas in normal CCA texture tends to be more hyperechoic, with lower contrast, more homogeneous, more smooth, less coarse and less distinctive, as shown in Figure 1.

**Table 1:** Statistical analysis of the texture features computed from 43 (15 normal and 28 apoE^−/−^) ultrasound images of mice common carotid arteries.

	Normal(n = 15)	ApoE^−/−^(n = 28)	p value	FDRcorrection
**First-Order Statistics (FOS)**				
AGL	197.8±13.4	136.9±25.8	0.0001	√
SD	22.42±3.78	25.98±4.35	0.009	√
**Fractal Dimension Texture Analysis**				
D*_f_*	2.022±0.067	1.996±0.029	0.169	NS
**Spatial Gray-Level Dependence Matrix(SGLDM)**				
CON	7.05±2.05	9.87±3.67	0.013	√
COR	0.89±0.039	0.904±0.028	0.228	NS
ENE	0.031±0.008	0.019±0.0048	0.0001	√
HOM	0.512±0.049	0.493±0.034	0.196	NS
ENT	1.347±0.065	1.437±0.058	0.0001	√
**Gray Level Difference Statistics (GLDS)**				
CON1	22.6±13.4	25.56±9.66	0.458	NS
ASM	0.171±0.043	0.138±0.026	0.015	√
ENT1	0.879±0.121	0.943±0.087	0.087	NS
MEAN	0.0142±0.003	0.016±0.0028	0.102	NS
**Neighborhood Gray Tone Difference Matrix(NGTDM)**				
COA	6.88±2.03	10.65±3.16	0.0001	√
CON2	0.455±0.13	0.623±0.304	0.057	NS
BUS	1.97±0.828e-5	1.39±0.66e-5	0.027	√
COM	6386±2395	9565±3667	0.001	√
STR	16571±8246	36144±19340	0.0001	√
**Statistical Feature Matrix(SFM)**				
CON3	1.176±0.107	1.202±0.116	0.477	NS
COV	0.74±0.067	0.86±0.102	0.0001	√

For each feature the mean and standard deviation were computed.

All features significant (p<0.05) after false discovery rate correction for multiple comparisons over all texture features.

**Figure 2:**
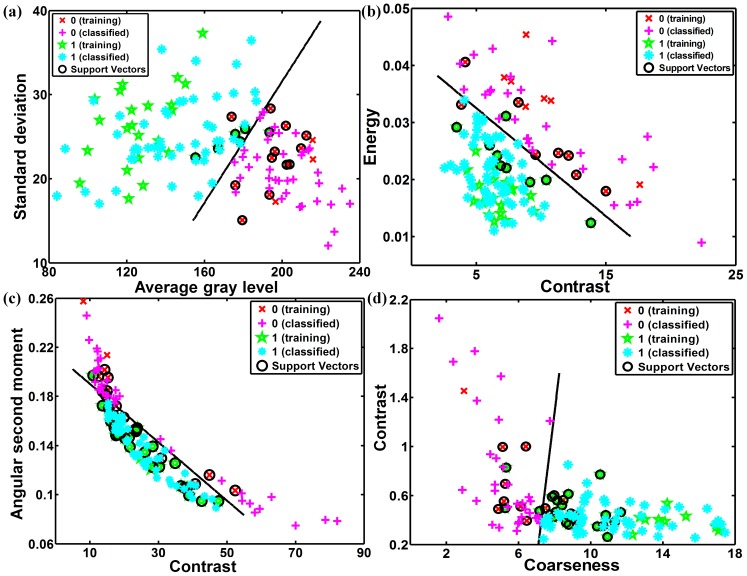
Two-dimensional scatter-plots showing training data, classified data, support vectors and decision boundaries for support vector machine classifier using (a) FOS, (b) SGLDM, (c) GLDS, (d) NGTDM.

For the classification task, the SVM classifier and the KNN classifier were implemented. Table 2 shows the classification accuracy, sensitivity and specificity obtained using these two classifiers, for different feature sets. For SVM classifier, the best feature set was the FOS with an accuracy of 83%, sensitivity of 80% and specificity of 90%, followed by the SGLDM with an accuracy of 80%, sensitivity of 85% and specificity of 70%, the NGTDM with an accuracy of 77%, sensitivity of 77% and specificity of 77%, and the GLDS with an accuracy of 71%, sensitivity of 73% and specificity of 67%. The worst feature set was SFM with an accuracy of 56%, sensitivity of 70% and specificity of 27%. In addition, when all 19 features were used for SVM classification, it reaches the accuracy, sensitivity and specificity of 87%, 88%, and 83%, respectively. When the 11 optimal features were chosen for SVM classification, it results in accuracy, sensitivity and specificity of 89%, 87%, 93%, respectively. Figure 2 shows two-dimensional scatter plots of SVM classifier using some of the features listed in Table 1. The figure shows that the two classes are well separated, and demonstrates that texture features can be used to identify arterial roughness accurately.

**Table 2:** The accuracy, sensitivity and specificity of the support vector machine and the k-nearest neighbor classifiers with *k* = 5 for five feature sets, for all the 19 features used as one feature set, and for the 11 optimal features shown in Table 1.

Feature Set	Feature SetVector Size	SVM Classifier			KNN Classifier		
		Accuracy	Sensitivity	Specificity	Accuracy	Sensitivity	Specificity
FOS	2	83%	80%	90%	90%	90%	87%
SGLDM	5	80%	85%	70%	62%	80%	27%
GLDS	4	71%	73%	67%	61%	67%	50%
NGTDM	5	77%	77%	77%	73%	75%	70%
SFM	2	56%	70%	27%	56%	70%	27%
All	19	87%	88%	83%	73%	75%	70%
Optimal	11	89%	87%	93%	73%	75%	70%

**Figure 3:**
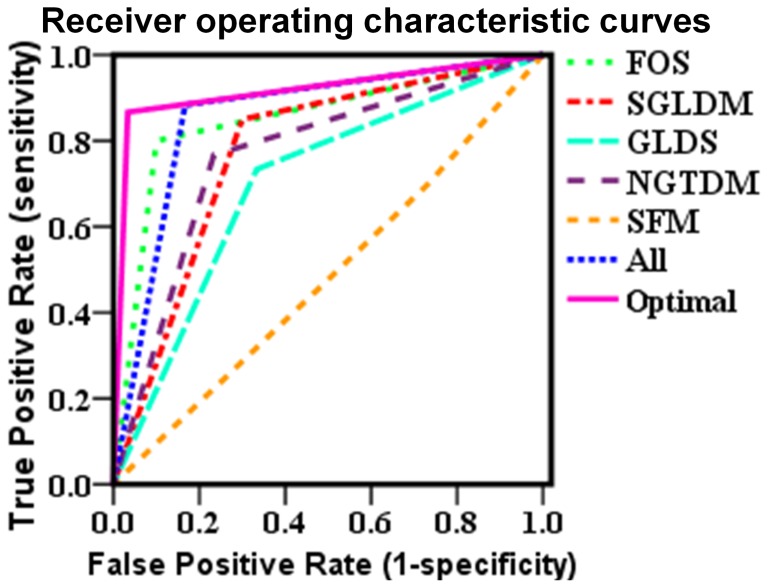
Receiver operating characteristic curves for support vector machine classifier with different feature sets.

The ROC analysis is a standard approach for evaluating the sensitivity and specificity of diagnostic procedures [36]. Figure 3 displays the ROC curves when different feature sets were used as inputs to the SVM classifier. The areas under the curves were calculated and presented in Table 3. The ROC curves and the areas show that the SGLDM and NGTDM can obtain a ROC area of 0.775 and 0.767, respectively. The FOS and all 19 features are better for identifying abnormal mice (AUROC: FOS = 0.850, All = 0.858). The optimal features selected can achieve maximum ROC area (0.900).

**Table 3:** Area under the Receiver operating characteristic curve for each feature set for support vector machine classifier.

Test Result Variables	Area
FOS	0.850
SGLDM	0.775
GLDS	0.700
NGTDM	0.767
SFM	0.483
All	0.858
Optimal	0.900

**Figure 4:**
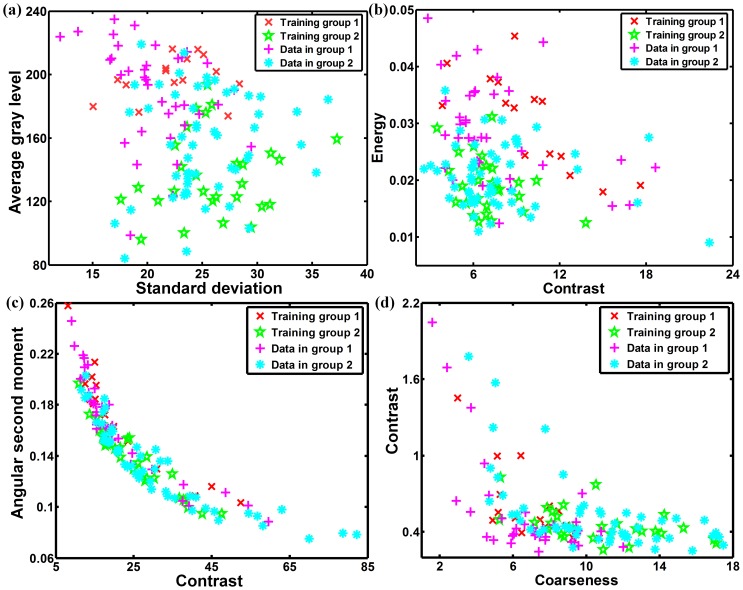
Two-dimensional scatter-plots showing training data and classified data for KNN classifier using (a) FOS, (b) SGLDM, (c) GLDS, (d) NGTDM.

**Figure 5:**
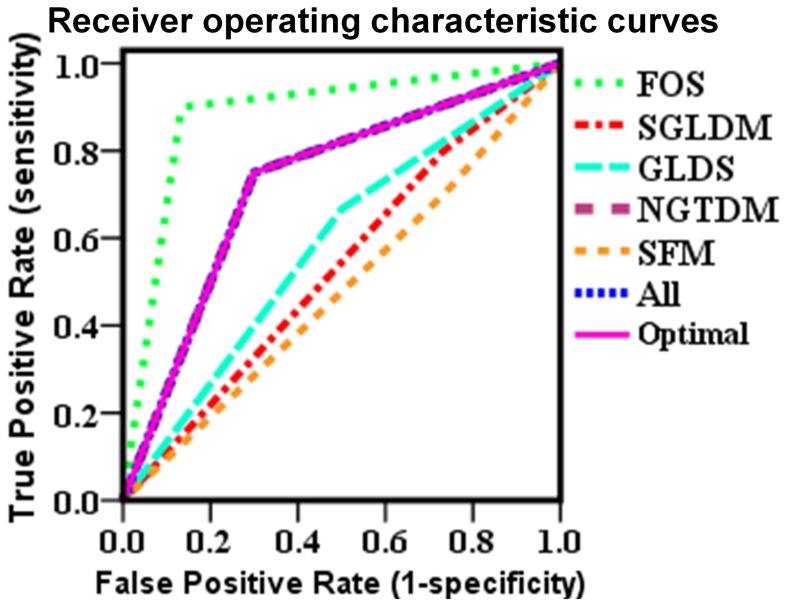
Receiver operating characteristic curves for k-nearest neighbor classifier with different feature sets.

The statistical KNN classifier was implemented and the results were compared with those of the SVM. In Table 2, the results are tabulated for *k = *5. The best individual result for the KNN classifier was also achieved with the FOS feature set with an accuracy of 90%, sensitivity of 90% and specificity of 87%, followed by the NGTDM with an accuracy of 73%, sensitivity of 75% and specificity of 70%, the SGLDM with an accuracy of 62%, sensitivity of 80% and specificity of 27%, and the GLDS with an accuracy of 61%, sensitivity of 67% and specificity of 50%. The worst feature set was SFM with an accuracy of 56%, sensitivity of 70% and specificity of 27%. When all 19 features or the 11 optimal features were used as one feature set, it comes out with the same accuracy, sensitivity and specificity at 73%, 75%, and 70%, respectively. Figure 4 shows two-dimensional scatter plots of KNN classifier using some of the features tabulated in Table 1. Compared with Figure 2, Figure 4 shows a higher degree of overlap of the texture features for the two classes. Figure 5 displays the ROC curves when different feature sets were used as inputs to the KNN classifier. The areas under the curves were calculated and presented in Table 4. The FOS is the best feature set for identifying abnormal mice, with ROC area of 0.883. NGTDM, all 19 features, and 11 optimal features all resulted in ROC areas of 0.725.

**Table 4:** Area under the Receiver operating characteristic curve for each feature set for k-nearest neighbor classifier.

Test Result Variables	Area
FOS	0.883
SGLDM	0.533
GLDS	0.583
NGTDM	0.725
SFM	0.483
All	0.725
Optimal	0.725

**Figure 6:**
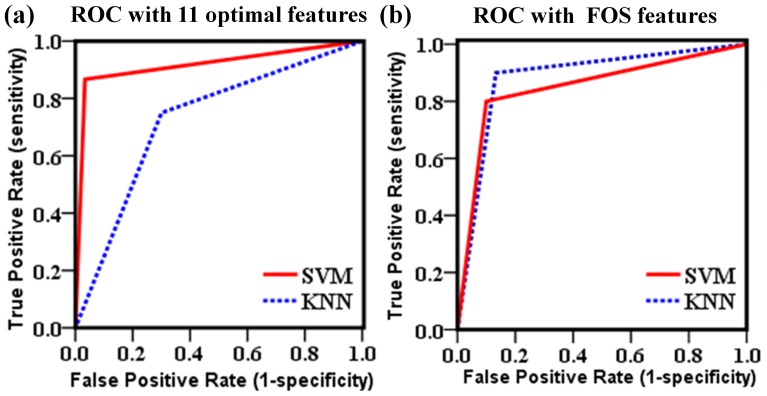
Receiver operating characteristic curves for the support vector machine and the k-nearest neighbor classifiers, using as input the 11 optimal feature sets (a) and first-order statistics feature set (b).

Figure 6 displays the ROC curves for the SVM and the KNN classifiers when 11 optimal features and only the FOS feature set were used to train the classifiers. With 11 optimal features used, SVM classifier showed better performance compared to the KNN classifier (AUROC: SVM = 0.900, KNN = 0.725), as shown in Figure 6(a). However, when the classifiers were trained with FOS feature set, the area below the curve was slightly higher for the KNN classifier, 0.883, whereas for the SVM classifier was 0.850, as shown in Figure 6(b).

**Figure 7:**
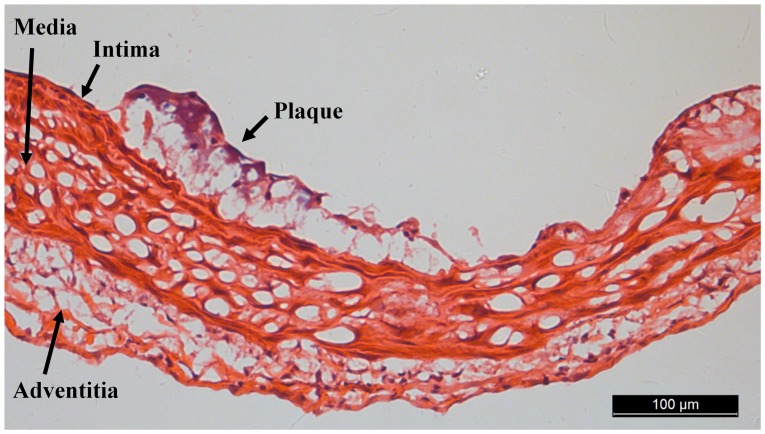
Photograph of atherosclerotic lesion in the common carotid artery of apoE^−/−^ mouse fed a high-fat diet with hematoxylin and eosin (H&E) staining.

Pathological evaluations of carotid lesions were carried out. Figure 7 shows the CCAs sections stained with H&E, which revealed the induction of atherosclerotic lesion formation.

## Discussion

This study aims to confirm the feasibility of using texture features for early identification of arterial roughness from ultrasound images, and to determine the optimal texture features. A total of 19 texture features were extracted from high-frequency ultrasonic images of mice carotid arteries. Statistical analysis indicated that 11 features can be used to distinguish between normal and abnormal groups, as shown in Table 1. Eleven optimal features, when combined in a feature set, achieved higher classification accuracy, sensitivity and specificity. These results are consistent with those of other studies [18], [37] and suggest that smooth surface, echogenicity, and a homogenous texture are characteristics of asymptomatic plaques, whereas irregular surface, echolucency and a heterogeneous texture are characteristics of potentially symptomatic plaques.

The SVM classifier and KNN classifier were implemented for the classification of mice with CCA abnormalities. The SVM classifier was chosen because it gives superior performance over other statistical approaches and because it is robust against overtraining and the curse of dimensionality [38]. The SVM is well suited for pattern classification problems, where there is a high degree of accuracy to identify arterial roughness, as shown in Figure 2. In this study, six different feature sets were considered at the beginning. However, the FDTA was proven to have no statistical significance and was therefore excluded. So, five different feature sets were extracted from the mice CCA images and used for training the SVM classifier. As shown in Table 2, the best feature sets was the FOS feature set, followed by the SGLDM, NGTDM, and the GLDS. In general, all feature sets performed in a range of about 71%–83%, excepting the SFM feature set that performed worse. When all the 19 features were used as inputs to the SVM classifier, it achieves over 87% accuracy, 88% sensitivity and 83% specificity. However, when the classifiers were trained with 11 optimal features, their classification performance improved to be 89% accuracy, 87% sensitivity and 93% specificity. The KNN classifier also performed well and yielded results comparable in most cases with the results obtained by the SVM classifier. The best individual result for the KNN classifier was also achieved with the FOS feature set, with an accuracy of 90%. As shown in Figure 6, using 11 optimal features as input, the SVM classifier showed better performance, while using FOS feature set as input, the KNN classifier achieved better performance. This suggests that different classifier designs potentially offer complementary information. As the previous work has shown [39], [40], hybrid SVM/KNN classifier taking the advantages of the SVM and KNN, could be harnessed to improve the overall classifier performance.

Carotid IMT is a well-accepted and commonly used surrogate marker for early atherosclerosis [41]. A variety of IMT measurement protocols, based on different ultrasound methods (B-mode and RF processing), have been developed and improved in order to obtain reliable and reproducible results [42], [43]. However, the B-mode method is time consuming and operator dependent [44]. The advantage of the method used in this study is that it is less time consuming and less operator dependent than the B-mode method. RF IMT method measures only the CCA segment of the far wall that is located 2 cm proximal to the origin of the carotid bulb [45]. The site of measurement of the RF approach may be less likely to contain plaques, because it is more distant from the bifurcation that is most prone to develop carotid plaques. The method used in this study can identify abnormalities at any position along the carotid artery. However, the study is still need for further studies and verification of clinical trials.

One of limitations of this study is that the B-mode images of mice CCA were not standardized. The quality of the ultrasound images affects the quality of the extracted texture features. Brightness and contrast of the B-mode image strongly depend on the system gain, time gain compensation and dynamic range [46]. Images obtained from different scanners, by different ultrasonographers and through different ultrasound systems at different settings may be different. Thus, images should be standardized to insure the measurements of the CCA echodensity to be comparable. In this study, the B-mode images of mice CCA were obtained using the Vevo 2100 high-resolution ultrasound system under the same conditions and by the same ultrasonographers, thus the standardization was not conducted in our study. However, this step may be necessary in order to get images with low intra- and inter-observer variability, which will allow the development of meaningful studies in relation to clinical events. In addition, studies have pointed out that it is not possible to extrapolate findings in the CCA to the bifurcation or the internal carotid artery, due to differences in geometry, flow pattern, cellularity and relationships with different risk factors [47]. Additional prospective studies will have to scrutinize whether the texture features of the CCA gives additional information with respect to the future risk of cardiovascular disease taking into consideration the complete atherosclerotic process of the carotid tree.

## Conclusions

In summary, the results in this study show that it is likely to identify early CCA abnormalities based on texture features extracted from high-resolution ultrasound images. The SVM classifier is able to diagnose the normal and abnormal CCA with an accuracy, sensitivity and specificity of 89%, 87%, 93%. This paves way for insight into the mechanisms of atherosclerosis and a more objective diagnosis methodology. Furthermore, it may be possible to identify and differentiate those individuals into high and low risk groups according to their cardiovascular risk.
